# The association of intravenous insulin and glucose infusion with intensive care unit and hospital mortality: a retrospective study

**DOI:** 10.1186/s13613-019-0507-x

**Published:** 2019-02-11

**Authors:** Sigrid C. van Steen, Saskia Rijkenberg, Peter H. J. van der Voort, J. Hans DeVries

**Affiliations:** 1 Department of Endocrinology, Amsterdam UMC, University of Amsterdam, Amsterdam, The Netherlands; 2 Department of Intensive Care, OLVG Hospital, P.O. Box 95500, 1090 HM, Amsterdam, The Netherlands; 3 TIAS, School for Business and Society, Tilburg University, Tilburg, The Netherlands

**Keywords:** Insulin, Glucose, Hyperglycemia, Glycemic control, Critical illness, Intensive care unit, Mortality

## Abstract

**Background:**

We assessed the association of intravenous insulin and glucose infusion with intensive care unit (ICU) and hospital mortality.

**Methods:**

For this retrospective association study, we used data from all patients admitted to a medical-surgical ICU between January 2012 and September 2017. We excluded patients admitted < 24 h, patients with a diabetic ketoacidosis, patients with a therapy restriction upon ICU admission and readmissions. Using multivariate logistic regression, we examined the relation between intravenous insulin and glucose infusion and ICU and hospital mortality for all patients. Additionally, we used the same model to analyze the outcomes for patients admitted > 72 h.

**Results:**

Of 9507 eligible patients, 3966 were included. After correction for potential confounders, intravenous insulin was associated with ICU and hospital mortality in patients admitted > 24 h (*n *= 3966) (odds ratio (OR) 1.09 [95% CI 1.05–1.13] and 1.09 [95% CI 1.06–1.13] per 0.1 IU/kg added, respectively). Likewise, intravenous glucose was associated with ICU mortality (OR 1.01 [95% CI 1.00–1.01]) but not with hospital mortality and (OR 1.00 [95% CI 1.00–1.01]) per g/day added, respectively. In patients admitted > 72 h (*n *= 1550), insulin dose was associated with both ICU and hospital mortality (*p *= 0.002 and *p *< 0.001, respectively), but glucose infusion was not (*p *= 0.08 and *p *= 0.2, respectively).

**Conclusions:**

Intravenous insulin administration is associated with an increased risk of ICU and hospital mortality, after correction for potential confounders. Parenteral glucose administration was limited in amount but was still associated with ICU mortality. However, based on these results, it is unknown whether this association is an epiphenomenon, or represents a true harm of insulin and glucose administration.

**Electronic supplementary material:**

The online version of this article (10.1186/s13613-019-0507-x) contains supplementary material, which is available to authorized users.

## Background

Stress-induced hyperglycemia is a common phenomenon in critically ill patients [1]. It is caused by an interplay of counterregulatory hormones and cytokines, resulting in beta cell secretory defects and insulin resistance [2–4]. The insulin resistance is characterized by a disproportionate hepatic glucose production, diminished muscular glucose uptake and increased lipolysis [5]. This hyperglycemia is associated with increased morbidity and mortality in various populations of critically ill patients [6–9]. The exact pathophysiological mechanism is as for now unknown, but studies have demonstrated that high plasma glucose levels increase inflammation and oxidation in vitro [10, 11]. Exogenous glucose, administered orally or intravenous, augments to the hyperglycemic state [12]. In a Dutch intensive care unit (ICU), intravenous glucose infusion was associated with ICU and hospital mortality, after correction for the mean blood glucose (BG) concentration [13]. From this, the hypothesis arose that glucose infusion per se should be minimalized, even without the presence of hyperglycemia.

Intravenous rapidly acting insulin is a highly effective glucose lowering drug and the standard treatment modality for hyperglycemia in the ICU [14]. Studies on insulin use in the ICU have focused mainly on the effect of insulin therapy with tight as compared to more liberal glucose targets [15–22]. However, these studies provided inconsistent evidence on the outcome benefits of strict BG lowering with insulin [23, 24]. Accordingly, current ICU guidelines recommend that insulin should be used to achieve intermediate BG targets [25, 26].

In general, insulin enhances glucose uptake in skeletal muscles and adipose tissue, stimulates glycogen and protein synthesis and inhibits gluconeogenesis, glycogenolysis, protein breakdown and lipolysis [27]. Non-metabolic actions include cell proliferation, enhancement of the immune response and anti-inflammatory effects by suppression of cytokine release [28–30]. In critical illness, there is evidence that certain biochemical pathways that are activated by insulin are associated with negative outcome [31]. Likewise, in type 2 diabetes patients, the relative safety of insulin therapy has been under discussion [32]. However, in those patients this discussion seems to be put to rest after proper correction for time-varying confounders [33]. Whether insulin infusion by itself is harmful in critically ill patients is to our knowledge unknown.

The effects of both intravenous glucose and insulin are entwined with overall glycemic control, making it difficult to draw conclusions on the separate effects. In a post hoc analysis of the Leuven study, the authors tried to address the observed outcome benefits to either glycemic control or insulin and concluded that achieving normoglycemia was most important, rather than the amount of infused insulin [34]. Altogether, for now it is unknown whether the beneficial effects of insulin therapy are to some extent independent of BG lowering or solely due to protection against the toxic effects of exogenous and endogenous glucoses. Thereby, the aim of the current study is to assess the association of intravenous insulin and glucose with ICU and hospital mortality.

## Methods

### Study design

This retrospective association study was conducted according to the principles of the Declaration of Helsinki [35] and in accordance with the Dutch medical Research Involving Human Subjects Act (WMO). The institutional review board of the OLVG, who waived the requirement for informed consent, approved the study protocol. We followed the Strengthening the Reporting of Observational studies in Epidemiology (STROBE) recommendations [36].

### Patients

All patients admitted to a 20-bed teaching hospital mixed medical-surgical ICU (OLVG Oost, Amsterdam, the Netherlands) between January 1, 2012, and September 29, 2017, were potentially eligible (*n *= 9507). We excluded patients who were admitted less than 24 h, since our ICU admits many elective cardiothoracic surgery patients, who are considered less severely ill. Moreover, we excluded patients with diabetic ketoacidosis and patients with a therapy restriction upon ICU admission. If a patient was readmitted at any point during the inclusion period, we considered data from the first admission. We analyzed all included patients (*n* = 3966) and patients admitted > 72 h (*n* = 1550) separately.

### Exposures and outcome

The main exposure variables of interest were intravenous insulin and glucose infusion. Individual patients were considered exposed to intravenous insulin or glucose when this was registered in the electronic patient data management system (PDMS, Meta Vision, *i*MD-Soft, Tel Aviv, Israel). Daily intravenous insulin dosage was calculated based on the hourly drip rate. All beds are equipped with a weighting scale, which was used for the bodyweight adjusted insulin dosage. Individual patients were considered exposed to intravenous glucose when they received glucose infusion in any form of glucose (in our ICU either glucose 5% or 20%). Daily intravenous glucose dosage was calculated in grams by summation of all glucose infusions (e.g., glucose 5% contains 50 mg/mL; and glucose 20% contains 200 mg/mL). In general, glucose 5% is used as volume resuscitation only when patients suffer from hypernatremia, or to dissolve certain medication. In other cases, volume resuscitation or maintenance was achieved with sodium chloride solutions. Glucose 20% is mainly administered as part of total parenteral feeding (TPV) or to treat hypoglycemia. Since there is a substantial difference in the net uptake of enteral glucose among individuals, and since plasma glucose is mainly dependent on glycogenolysis and gluconeogenesis, we did not take account of enteral feeding in the total amount of intravenous infused glucose. Outcomes of interest were all-cause ICU and hospital mortality. We followed patients until death or discharge from the ICU or the hospital.

### Standard ICU care

BG control was executed by the nursing staff following unit guidelines based on intermittent measurements with a handheld point-of-care device (Accu-Chek, Roche, Switzerland). Short-acting insulin (NovoRapid^®^, Novo Nordisk, Bagsvӕrd, Denmark) was continuously infused with a syringe pump. This was guided by a validated computerized dynamic sliding-scale algorithm integrated in the PDMS [37]. The BG regulation protocol started when a patient had one BG measurement > 10 mmol/L and targeted a BG concentration of 5.0–9.0 mmol/L. This BG target changed during the study period of 6.0–9.0 mmol/L. Routinely, enteral feeding was started as soon as possible. Caloric needs were calculated based on eight times the mean VCO_2_ output (mL/min) measured over the last 24 h or shorter as data were available. Enteral feeding was continuously administered by nasogastric tube, started at a rate of 20 mL/h. Rate increased every 6 h until the target was reached. On the day of admission, the target was 25% of the caloric needs, increasing by 25% every day. Gastric residuals were evaluated every 6 h. Parenteral nutrition was given as separate components and administered as soon as possible when a patient had contraindication for enteral feeding (e.g., gastrointestinal failure) or when the protein goals are not met. All patients received a single dose of steroids on admission (dexamethasone, 1 mg/kg with a maximum of 100 mg), except from when they already received this at the operation or emergency room. Steroids during admission administered on indication were prednisolone, methylprednisolone, dexamethasone or hydrocortisone.

### Data collection

All data were extracted retrospectively from the PDMS and analyzed from September 1, 2017, to December 1, 2017. Extracted data were encoded; the key was saved in a different dataset, stored in different locations and only available for the involved investigators. Besides the exposure and outcome data, collected variables included demographic and admission-related characteristics.

### Statistical analysis

Continuous variables are presented as mean with standard deviation (SD) or median with interquartile range (IQR), depending on their distribution. Continuous variables were examined for normality using the Shapiro–Wilk test. Nominal variables are presented as number with proportion. Patient characteristics were compared between survivors and non-survivors (based on ICU discharge status) with a Chi-square, independent-sample *t*, Mann–Whitney *U*, ANOVA or Kruskal–Wallis test, depending on the data distribution.

The primary analysis evaluated the association between the amount of intravenous insulin or glucose (continuous and categorized) and ICU and hospital mortality. Given the dichotomous outcome, a multivariable logistic regression model was used and outcomes were reported as odds ratios (ORs) with a 95% confidence interval. The category with no insulin or glucose infusion is used as a reference. We based our choice of covariates on previous research and our idea of clinically relevant confounders [38–40]. Hereby, we chose to correct for variables that potentially confound the relation between insulin, glucose and mortality, such as severity of disease, age, prehospital diabetes status, use of corticosteroids, feeding and glycemic measures. Using a combination of backward elimination and forward selection, we determined the appropriate parameters for the model [41]. Because of the possible overlap between different severity of disease scores and different glycemic measures, we checked for collinearity using the variance inflation factor (VIF). For each continuous covariate, we tested the magnitude of the association across the range of values for that variable. We included the interaction between insulin and glucose infusion in the analysis to ensure that the estimated associations were valid. Additionally, we analyzed the association between intravenous insulin and glucose in patients admitted > 72 h and compared results.

Missing covariate data were replaced with the use of multiple imputations [the Markov chain Monte Carlo (MCMC) method]. We made no adjustment for multiple comparisons. Two-sided *p* values < 0.05 were considered significant. All statistical analyses were performed using SPSS version 23 (SPSS Corp., Chicago, IL).

## Results

### Participants

**Fig. 1:**
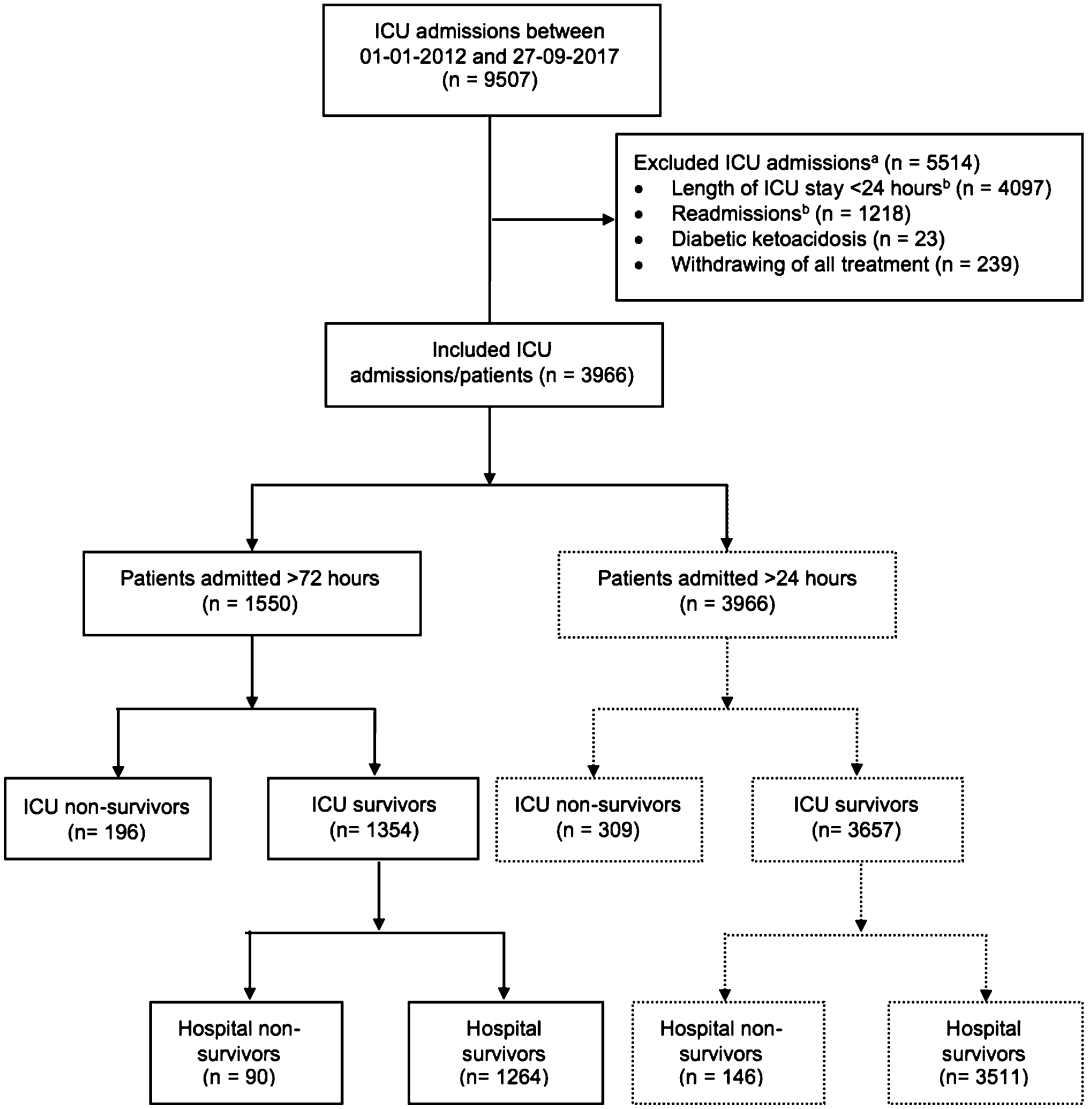
Patient flow diagram. ^a^Category numbers do not sum up due to overlap between exclusion criteria. ^b^Readmissions are defined as all ICU admissions after the initial admission. *ICU* intensive care unit

There were 9507 ICU admissions between January 1, 2012, and September 27, 2017 (Fig. 1). After exclusion of admissions with a duration less than 24 h, readmissions, patients with a diabetic ketoacidosis and patients with a treatment restriction upon ICU admission, 3966 patients remained. Of all included patients (*n *= 3966), 309 (7.8%) died in the ICU and 455 (11.5%) in the hospital. Of patients admitted > 72 h (*n* = 1550), 196 (12.6%) died in the ICU and 286 (18.5%) died in the hospital.

### Baseline and admission-related characteristics

Table 1 shows the baseline and admission-related characteristics for the total cohort and for patients admitted > 72 h, comparing ICU survivors with non-survivors. With regard to the cohort of patients admitted > 72 h, the mean age was 70 years and the cohort consisted of more males (63.4%). Almost 90% of the patients received steroids during their ICU admission. Over 20% of the patients had a history of diabetes, and half of these patients previously used insulin. The non-survivors were significantly more often admitted for medical conditions (74% vs. 60%), while the survivor cohort consisted of more, especially elective (cardiothoracic), surgery patients (28% vs. 12%). Non-survivors had higher severity of disease scores [Acute Physiology and Chronic Health Evaluation IV (APACHE) and Sequential Organ Failure Assessment (SOFA)] and were consequently more often in need of mechanical ventilation, renal replacement therapy and had a longer duration of stay in the ICU. They had more low BG measurements (< 4.4 mmol/L). Almost 90% of all patients received intravenous insulin during admission. The median amount per day was higher in non-ICU survivors (39.3 IU) as compared to ICU survivors (27.3 IU) (*p* < 0.001). Over three-quarter of patients received intravenous glucose. Likewise, the median amount per day was higher in non-ICU survivors (3.5 vs 1.1 g, *p* < 0.001). Additional file 1: Tables S1 and S2 give information on the main indications for glucose infusion in those patients. Patients who received glucose 5% more frequently suffered from hypernatremia. Of the patients receiving 20%, 30% received this as part of total parenteral feeding, and 70% experienced at least one incidence of a BG measurement < 4.4 mmol/L. Over half of the intake in kilocalories was provided by enteral feeding, the rest was provided by either oral intake, or infusion of glucose containing solutions. The amount of enteral feeding was equal for survivors and non-survivors from admission day 2.

**Table 1:** Baseline and admission-related characteristics for the total cohort of included patients admitted (*n *= 3966) and for the cohort of patients admitted > 72 h (*n *= 1550), comparing ICU survivors with non-survivors (*n *= 196)

Variable	All patients				Admitted > 72 h			
	ICU survivors (*n *= 3695)	ICU non-survivors (*n *= 271)	*p* value^a^	All (*n* = 3966)	ICU survivors (*n *= 1354)	ICU non-survivors (*n *= 196)	*p* value^a^	All > 72 h (*n *= 1550)
Age (years)	68.8 ± 13.7	70.3 ± 12.7	0.05	68.9 ± 13.6	69.0 ± 13.2	71.4 ± 12.0	0.019	69.3 ± 13.1
Gender (male)	2377 (64.3)	161 (59.4)	0.11	2538 (64.0)	861 (63.6)	122 (62.2)	0.775	983 (63.4)
Body mass index (kg/m^2^)	27.1 ± 6.2	26.9 ± 5.4	0.59	26.9 ± 5.4	27.2 ± 6.0	27.0 ± 6.1	0.640	27.1 ± 6.0
History of diabetes^b^	771 (20.8)	66 (24.4)	0.19	837 (21.1)	304 (22.5)	44 (22.4)	1.000	348 (22.5)
Insulin dependent	315 (8.5)	35 (12.9)	0.02	350 (8.8)	139 (10.3)	22 (11.2)	0.775	161 (10.4)
Oral anti-diabetes medication	547 (14.8)	37 (13.7)	0.66	584 (14.7)	203 (15.0)	24 (12.2)	0.363	227 (14.6)
History of liver cirrhosis	57 (1.5)	8 (3.0)	0.08	65 (1.6)	21 (1.6)	3 (1.5)	1.000	24 (1.5)
Type of admission^c^			< 0.001				< 0.001	
Medical	1680	211		1891 (47.7)	814 (60.1)	144 (73.5)		958 (61.8)
Elective/scheduled surgery	1690 (45.7)	27 (10)		1717 (43.3)	385 (28.4)	24 (12.2)		409 (26.4)
Urgent/emergency surgery	325 (8.8)	33 (12.2)		358 (9.0)	155 (11.4)	28 (14.3)		183 (11.8)
Cardiothoracic surgery patients	1715 (46.4)	33 (1.2)	< 0.001	1748 (44.1)	428 (31.6)	26 (13.3)	< 0.001	454 (29.3)
Severity of disease scores								
APACHE IV PM (%)	19.6 [2.4 to 28.4]	60.4 [40.0 to 84.4]	< 0.001	22.4 [2.7 to 34.3]	22.4 [8.0 to 47.9]	50.7 [29.3 to 80.0]	< 0.001	25.2 [9.3 to 53.0]
SOFA score on admission	6.4 ± 2.7	10.7 ± 3.9	< 0.001	6.7 ± 3.0	7.7 ± 3.0	9.9 ± 3.6	< 0.001	8.0 ± 3.1
Maximum SOFA score	6.7 ± 2.9	12.8 ± 4.0	< 0.001	7.1 ± 3.4	8.5 ± 3.1	12.4 ± 3.9	< 0.001	9.0 ± 3.4
Mechanical ventilation	3102 (84.0)	263 (97)	< 0.001	3365 (84.8)	1238 (91.4)	190 (96.9)	0.011	1428 (92.1)
Renal replacement therapy	351 (9.9)	129 (47.6)	< 0.001	480 (12.1)	295 (21.8)	92 (46.9)	< 0.001	387 (25.0)
Corticosteroids during admission	2555 (69)	248 (91.5)	< 0.001	2803 (70.7)	1199 (88.6)	183 (93.4)	0.057	1382 (89.2)
Sodium, average > 145 mmol/L	160 (4.3)	34 (12.5)	< 0.001	194 (4.9)	113 (8.3)	30 (15.3)	0.003	143 (9.2)
Total parenteral feeding	90 (2.4)	21 (7.7)	0.19	111 (2.8)	70 (5.2)	21 (10.7)	0.968	91 (5.9)
Daily intake (kcal)	678 [339 to 1177]	1136 [658 to 1495]	< 0.001	705 [357 to 1214]	1300 [974 to 1619]	1368 [1111 to 1676]	0.056	1312 [985 to 1630]
Daily intake form enteral feeding (kcal)	315 [0 to 880]	830 [467 to 1278]	< 0.001	100 [0 to 717]	790 [49 to 1174]	864 [475 to 1171]	0.039	803 [60 to 1173]
Admission day 1 (kcal)	0 [0 to 288]	279 [95 to 530]	< 0.001	0 [0 to 308]	171 [0 to 435]	296 [56 to 543]	0.001	185 [0 to 448]
Admission day 2 (kcal)	314 [0 to 937]	818 [463 to 1199	< 0.001	387 [0 to 959]	872 [477 to 1199]	924 [596 to 1248]	0.320	881 [480 to 1199]
Admission day 3 (kcal)	480 [0 to 1224]	1017 [510 to 1439]	< 0.001	588 [0 to 1247]	1199 [802 to 1508]	1199 [847 to 1507]	0.964	1199 [808 to 1506]
Length of ICU stay (h)	51 [38 to 115]	120 [54 to 244]	< 0.001	55.0 [38.0 to 119.0]	143 [99 to 261]	190 [121 to 330]	< 0.001	148 [102 to 269]
Mean BG (mmol/L)	8.3 ± 1.2	7.9 ± 1.3	< 0.001	8.3 ± 1.2	8.0 ± 1.0	8.0 ± 0.9	0.606	8.0 ± 1.0
MAG change (mmol/L/h)	0.36 [0.22 to 0.57]	0.43 [0.28 to 0.60]	0.001	0.4 [0.2 to 0.6]	0.3 [0.2 to 0.5]	0.4 [0.3 to 0.5]	0.007	0.4 [0.2 to 0.5]
Time in glycemic ranges								
BG percentage < 4.4 mmol/L	0.0 [0.0 to 0.0]	0.0 [0.0 to 3.0]	< 0.001	0.0 [0.0 to 0.0]	0.0 [0.0 to 0.6]	0.0 [0.0 to 2.2]	< 0.001	0.0 [0.0 to 1.0]
BG percentage 4.4–5.9 mmol/L	5.4 [0.0 to 13.7]	9.5 [3.6 to 20.0]	< 0.001	5.7 [0.0 to 14.3]	8.7 [4.3 to 14.9]	8.8 [4.1 to 16.7]	0.878	8.7 [4.3 to 15.0]
BG percentage 6.0–9.0 mmol/L	61.1 [45.5 to 75.0]	58.0 [42.9 to 70.0]	0.006	60.9 [45.5 to 75.0]	64.1 [51.5 to 75.0]	62.9 [50.0 to 70.6]	0.340	63.9 [51.3 to 74.5]
BG percentage 9.1–11.1 mmol/L	20.8 [10.0 to 31.0]	17.4 [8.6 to 25.8]	< 0.001	20.3 [9.9 to 30.8]	18.9 [9.1 to 26.9]	18.8 [11.7 to 25.9]	0.939	18.8 [9.6 to 26.8]
BG percentage > 11.1 mmol/L	3.0 [0 to 14.3]	4.2 [0 to 11.9]	0.22	3.1 [0.0 to 13.7]	3.7 [0.0 to 10.7]	4.8 [0.0 to 10.9]	0.221	3.9 [0.0 to 10.7]
Incidence BG < 2.2 mmol/L^e^	15 (0.4)	6 (2.2)	< 0.001	21 (0.5)	11 (0.8)	4 (2.0)	0.211	15 (1.0)
Incidence BG < 4.4 mmol/L^e^	535 (14.5)	120 (44.3)	< 0.001	655 (16.5)	371 (27.4)	88 (44.9)	< 0.001	459 (29.6)
Intravenous insulin (IU)								
Number of patients	2931 (79.3)	237 (87.5)	0.001	3168 (79.9)	1205 (89.0)	184 (93.9)	0.049	1389 (89.6)
Total amount during admission	64 [15 to 187]	216 [60 to 530]	< 0.001	69.0 [17.0 to 205.0]	215 [80 to 483]	398 [171 to 834]	< 0.001	232 [85 to 514]
Amount per day	18.7 [4.8 to 39.3]	34.4 [12 to 60.1]	< 0.001	19.5 [5.2 to 40.7]	27.3 [11.4 to 53.8]	39.9 [22.9 to 64.1]	< 0.001	29.0 [12.2 to 55.1]
Amount per kg per day^f^	0.24 [0.06 to 0.48]	0.43 [0.17 to 0.77]	< 0.001	0.3 [0.1 to 0.5]	0.3 [0.1 to 0.6]	0.5 [0.3 to 0.8]	< 0.001	0.4 [0.2 to 0.7]
Intravenous glucose (g)								
Number of patients	1674 (45.3)	233 (86.0)	< 0.001	1907 (48.1)	993 (73.3)	177 (90.3)	< 0.001	1170 (75.5)
Total amount during admission	0.0 [0.0 to 7.8]	27.1 [5.1 to 96.7]	< 0.001	0.0 [0.0 to 10.4]	8.7 [0.0 to 33.9]	38.4 [11.7 to 103.5]	< 0.001	10.2 [0.5 to 40.6]
Amount per day	0.0 [0.0 to 1.4]	3.2 [1.2 to 11.9]	< 0.001	0.0 [0.0 to 1.8]	1.1 [0.0 to 3.3]	3.5 [1.5 to 9.0]	< 0.001	1.3 [0.1 to 3.9]
Fluid balance (L)	2.2 [0.46 to 4.3]	9.3 [4.4 to 17.7]	< 0.001	2.4 [0.6 to 4.7]	2.1 [− 0.5 to 5.4]	8.6 [4.4 to 18.0]	< 0.001	2.7 [− 0.2 to 6.4]

Data are presented as mean ± standard deviation, median [interquartile range] or number (%). Percentages might not sum up to 100 due to rounding

*APACHE* Acute Physiological and Chronic Health Evaluation, *BG* blood glucose, *ICU* intensive care unit, *MAG* mean absolute glucose, *SOFA* Sequential Organ Failure Assessment

^a^*P* values are based on the Student’s *t* test or the Mann–Whitney *U* test for continuous data, and on the Chi-square test (with continuity correction), ANOVA or Kruskal–Wallis for categorical data, comparing ICU survivors with ICU non-survivors

^b^Based on the use of any diabetes medication before admission

^c^Based on the Intensive Care National Audit and Research Centre (ICNARC) model

^d^BG data are based on point-of-care measurements with the handheld Accu-Chek device

^e^Number of patients with at least one BG measurement < 2.2 mmol/L or < 4.4 mmol/L

^f^Weight is based on the admission weight as measured by the bed weighting scale

The cohort admitted > 72 h had less elective (cardiothoracic) surgery patients (29% vs 44%), and the overall severity of disease score was more or less the same (APACHE IV-predicted mortality 25.2% vs 22.4%). The > 72 h cohort used more intravenous insulin and glucose.

### Main results

We analyzed all included patients (*n* = 3966) and the patients with a length of stay > 72 h separately. In the complete cohort, a history of insulin use and liver cirrhosis, as well as mean BG and BG variability [expressed as mean absolute glucose (MAG) change], was associated with a higher mortality risk (Table 2).

**Table 2:** Univariate logistic regression analysis for the association with ICU and hospital mortality in all included patients (*n* = 3966)

Variable	ICU mortality (*n* = 309)		Hospital mortality (*n* = 455)	
	OR (95% CI)	*p* value	OR (95% CI)	*p* value
Age (per 10 years)	1.14 (1.04, 1.25)	0.006	1.26 (1.16, 1.36)	< 0.001
Gender (male as reference)	1.19 (0.94, 1.51)	0.148	1.15 (0.94, 1.41)	0.172
BMI (per kg/m^2^)	1.01 (0.99, 1.03)	0.438	1.00 (0.98, 1.01)	0.577
History of diabetes	1.19 (0.91, 1.57)	0.203	1.27 (1.01, 1.60)	0.039
History of insulin use	1.55 (1.09, 2.22)	0.015	1.50 (1.10, 2.04)	0.010
History of liver cirrhosis	2.46 (1.27, 4.76)	0.007	3.81 (2.25, 6.47)	< 0.001
Cardiothoracic surgery patients	0.22 (0.16, 0.30)	< 0.001	0.16 (0.12, 0.21)	< 0.001
APACHE IV PM (per %)	1.04 (1.04, 1.05)	< 0.001	1.04 (1.04, 1.05)	< 0.001
Maximum SOFA score (per point)	1.50 (1.45, 1.56)	< 0.001	1.41 (1.36, 1.45)	< 0.001
Corticosteroids during admission	3.88 (2.67, 5.63)	< 0.001	3.28 (2.46, 4.38)	< 0.001
Average sodium > 145 mmol/L	3.26 (2.25, 4.74)	< 0.001	3.53 (2.55, 4.89)	< 0.001
Total parenteral feeding	0.79 (0.47, 1.33)	0.370	0.80 (0.49, 1.30)	0.359
Daily intake (all sources, per 100 kcal)	1.02 (1.01, 1.03)	< 0.001	1.03 (1.02, 1.03)	< 0.001
Length of ICU stay (per day)	1.04 (1.03, 1.05)	< 0.001	1.04 (1.03, 1.05)	< 0.001
Mean BG (per mmol/L)	0.80 (0.72, 0.89)	< 0.001	0.81 (0.75, 0.88)	< 0.001
MAG change (per mmol/L/h)	1.48 (1.03, 2.11)	0.034	1.32 (0.97, 1.80)	0.083
Percentage BG < 4.4 mmol/L (per %)	1.07 (1.05, 1.09)	< 0.001	1.07 (1.05, 1.09)	< 0.001
Percentage BG > 11.1 mmol/L (per %)	1.00 (0.99, 1.01)	0.649	1.00 (0.99, 1.01)	0.613
Incidence glucose < 2.2 mmol/L	4.81 (1.85, 12.48)	0.001	3.11 (1.20, 8.07)	0.019
Incidence glucose < 4.4 mmol/L	3.88 (3.04, 4.97)	< 0.001	3.51 (2.83, 4.34)	< 0.001
IV insulin per kg per day (per 0.1 IU)	1.10 (1.07, 1.12)	< 0.001	1.10 (1.08, 1.12)	< 0.001
IV glucose per day (per g increase)	1.01 (1.01, 1.02)	< 0.001	1.01 (1.01, 1.02)	< 0.001
Fluid balance (per L increase)	1.22 (1.19, 1.24)	< 0.001	1.17 (1.15, 1.19)	< 0.001

For categorical variables, no was used as reference category

*APACHE* Acute Physiology and Chronic Health Evaluation, *BG* blood glucose, *BMI* body mass index, *CI* confidence interval, *ICU* intensive care unit, *IU* international unit, *IV* intravenous, *MAG* mean absolute glucose, *OR* odds ratio

The following variables are independently associated with ICU and hospital mortality in multiple regression analysis (Table 3): age, APACHE IV PM, maximal SOFA score, hypernatremia (average sodium > 145 mmol/L), percentage BG measurements < 4.4 mmol/L and the amount of intravenous insulin and fluid balance. Glucose intake increased the risk of ICU mortality and showed a trend toward hospital mortality. We found no interaction between insulin and glucose infusion on ICU mortality. The mean BG concentration had a protective effect on both ICU and hospital mortality (OR 0.83 [95% CI 0.70–0.99] and OR 0.76 [95% CI 0.66–0.88], respectively). A higher daily intake and cardiac surgery patients were protective too.

**Table 3:** Multivariate logistic regression analysis for the association with ICU and hospital mortality in all included patients (*n* = 3966)

Variable	ICU mortality (*n* = 309)		Hospital mortality (*n* = 455)	
	Adjusted OR (95% CI)	*p* value	Adjusted OR (95% CI)	*p* value
Age (per 10 years)	1.21 (1.07, 1.36)	0.002	1.38 (1.25, 1.52)	< 0.001
Cardiothoracic surgery patients	0.61 (0.39, 0.96)	0.032	0.34 (0.23, 0.49)	< 0.001
APACHE IV PM (per %)	1.02 (1.01, 1.03)	< 0.001	1.02 (1.01, 1.03)	< 0.001
Maximum SOFA score (per point)	1.26 (1.19, 1.34)	< 0.001	1.18 (1.13, 1.24)	< 0.001
Average sodium > 145 mmol/L	2.09 (1.29, 3.40)	0.003	2.17 (1.44, 3.27)	< 0.001
Daily intake (from all sources, per 100 kcal)	0.92 (0.89, 0.95)	< 0.001	0.94 (0.91, 0.97)	< 0.001
Mean BG (per mmol/L)	0.83 (0.70, 0.99)	0.040	0.76 (0.66, 0.88)	< 0.001
Percentage BG < 4.4 mmol/L (per %)	1.02 (1.00, 1.05)	0.089	1.02 (1.00, 1.05)	0.050
IV insulin per kg per day (per 0.1 IU)	1.09 (1.05, 1.13)	< 0.001	1.09 (1.06, 1.13)	< 0.001
IV glucose per day (per g increase)	1.01 (1.00, 1.01)	0.021	1.00 (1.00, 1.01)	0.094
Fluid balance (per L increase)	1.11 (1.08, 1.13)	< 0.001	1.08 (1.06, 1.11)	< 0.001

For categorical variables, ‘no’ was used as reference category

*APACHE* Acute Physiology and Chronic Health Evaluation, *BG* blood glucose, *BMI* body mass index, *CI* confidence interval, *ICU* intensive care unit, *IU* international unit, *IV* intravenous, *MAG* mean absolute glucose, *OR* odds ratio

Table 4 shows the result of the univariate analyses for the cohort of patients admitted > 72 h. A higher age, higher APACHE and maximum SOFA score, a longer stay at the ICU and the administration of intravenous insulin, intravenous glucose, steroids and a higher fluid load were significantly associated with ICU and hospital mortality. Likewise, the occurrence of hypernatremia and low BG measurements were associated with mortality. Patients who were admitted for cardiothoracic surgery had a lower mortality risk. The severity of disease scores (and length of stay) as well as the glycemic measures had a VIF value lower than three, indicating low collinearity levels.

**Table 4:** Univariate logistic regression analysis for the association with ICU and hospital mortality in the cohort of patients admitted > 72 h (*n* = 1550)

Variable	ICU mortality (*n* = 196)		Hospital mortality (*n* = 286)	
	OR (95% CI)	*p* value	OR (95% CI)	*p* value
Age (per 10 years)	1.16 (1.02, 1.31)	0.019	1.30 (1.16, 1.45)	< 0.001
Gender (male as reference)	1.06 (0.78, 1.44)	0.715	0.96 (0.73, 1.26)	0.768
BMI (per kg/m^2^)	0.99 (0.97, 1.02)	0.640	0.98 (0.96, 1.01)	0.155
History of diabetes	1.00 (0.70, 1.43)	0.999	1.26 (0.94, 1.70)	0.127
History of insulin use	1.11 (0.69, 1.78)	0.681	1.18 (0.79, 1.77)	0.424
History of liver cirrhosis	0.99 (0.29, 3.34)	0.983	2.29 (0.97, 5.39)	0.059
Cardiothoracic surgery patients	0.47 (0.32, 0.69)	< 0.001	0.33 (0.23, 0.47)	< 0.001
APACHE IV PM (per %)	1.03 (1.02, 1.03)	< 0.001	1.03 (1.02, 1.03)	< 0.001
Maximum SOFA score (per point)	1.38 (1.31, 1.44)	< 0.001	1.29 (1.24, 1.35)	< 0.001
Corticosteroids during admission	1.82 (1.01, 3.27)	0.046	1.73 (1.07, 2.81)	0.027
Average sodium > 145 mmol/L	1.99 (1.29, 3.06)	0.002	2.10 (1.43, 3.08)	< 0.001
Total parenteral feeding	0.95 (0.53, 1.70)	0.849	0.95 (0.55, 1.63)	0.851
Daily intake (from all sources, per 100 kcal)	1.01 (0.99, 103)	0.448	1.01 (0.99, 1.03)	0.209
Length of ICU stay (per day)	1.02 (1.00, 1.03)	0.009	1.02 (1.01, 1.03)	0.003
Mean BG (per mmol/L)	0.96 (0.83, 1.12)	0.606	0.97 (0.85, 1.11)	0.675
MAG change (per mmol/L/h)	1.32 (0.74, 2.36)	0.349	1.39 (0.84, 2.29)	0.198
Percentage BG < 4.4 mmol/L (per %)	1.09 (1.05, 1.13)	< 0.001	1.09 (1.05, 1.13)	< 0.001
Percentage BG > 11.1 mmol/L (per %)	1.00 (0.98, 1.01)	0.782	1.00 (0.99, 1.02)	0.722
Incidence BG < 2.2 mmol/L	2.54 (0.80, 8.07)	0.113	1.64 (0.52, 5.20)	0.397
Incidence BG < 4.4 mmol/L	2.16 (1.59, 2.93)	< 0.001	2.03 (1.56, 2.65)	< 0.001
IV insulin per kg per day (per 0.1 IU)	1.07 (1.04, 1.10)	< 0.001	1.07 (1.05, 1.10)	< 0.001
IV glucose per day (per g increase)	1.01 (1.00, 1.01)	< 0.001	1.01 (1.00, 1.01)	< 0.001
Fluid balance (per L increase)	1.14 (1.12, 1.17)	< 0.001	1.11 (1.09, 1.14)	< 0.001

For categorical variables (yes/no), no was used as reference category

*APACHE* Acute Physiology and Chronic Health Evaluation, *BG* blood glucose, *BMI* body mass index, *CI* confidence interval, *ICU* intensive care unit, *IU* international unit, *IV* intravenous, *MAG* mean absolute glucose, *OR* odds ratio

Table 5 shows the odds ratios (ORs) for ICU and hospital mortality after correction for confounders. In this cohort, the following variables are associated with ICU and hospital mortality: age, APACHE IV PM, maximal SOFA score, hypernatremia, percentage BG measurements < 4.4 mmol/L and the amount of intravenous insulin increased the risk of mortality. With regard to ICU mortality, the OR for intravenous insulin was 1.06 per 0.1 IU/day added (95% CI 1.02–1.09). A higher glucose load per day showed a trend toward higher ICU mortality with an OR of 1.01 per g/day added (95% CI 1.00–1.01; *p *= 0.08). We found no interaction between insulin and glucose infusion on ICU mortality. Additional file 1: Table S3 shows the adjusted ORs for ICU and hospital mortality per stratum of intravenous insulin administration (with no insulin as a reference group). The ORs increase linearly as insulin dose rose, but groups are not significantly different. Likewise, Additional file 1: Table S4 shows the results for intravenous glucose.

**Table 5:** Multivariate logistic regression analysis for the association with ICU and hospital mortality in the cohort of patients admitted > 72 h (*n* = 1550)

Variable	ICU mortality (*n* = 196)		Hospital mortality (*n* = 286)	
	Adjusted OR (95% CI)	*p* value	Adjusted OR (95% CI)	*p* value
Age (per 10 years)	1.25 (1.07, 1.46)	0.005	1.43 (1.25, 1.64)	< 0.001
Cardiothoracic surgery patients	0.68 (0.39, 1.18)	0.167	0.46 (0.28, 0.73)	0.001
APACHE IV PM (per %)	1.01 (1.00, 1.02)	0.010	1.01 (1.01, 1.02)	< 0.001
Maximum SOFA score (per point)	1.23 (1.15, 1.31)	< 0.001	1.15 (1.08, 1.21)	< 0.001
Average sodium > 145 mmol/L	2.00 (1.18, 3.40)	0.010	1.97 (1.25, 3.12)	0.004
Daily intake (from all sources, per 100 kcal)	0.91 (0.87, 0.96)	< 0.001	0.95 (0.91, 0.99)	0.007
Percentage BG < 4.4 mmol/L (per %)	1.06 (1.01, 1.12)	0.025	1.07 (1.02, 1.12)	0.003
IV insulin per kg per day (per 0.1 IU)	1.06 (1.02, 1.09)	0.002	1.06 (1.03, 1.09)	< 0.001
IV glucose per day (per g increase)	1.01 (1.00, 1.01)	0.083	1.00 (1.00, 1.01)	0.204
Fluid balance (per L increase)	1.10 (1.07, 1.12)	< 0.001	1.08 (1.05, 1.10)	< 0.001

For categorical variables, ‘no’ was used as reference category

*APACHE* Acute Physiology and Chronic Health Evaluation, *BG* blood glucose, *BMI* body mass index, *CI* confidence interval, *ICU* intensive care unit, *IU* international unit, *IV* intravenous, *OR* odds ratio

## Discussion

With this study, we explored the association between intravenous insulin and glucose infusion and ICU and hospital mortality. In this mixed population of critically ill patients, the amount of intravenous insulin and glucose infusion was both associated with ICU mortality after correction for confounders. Glucose infusion was not related to hospital mortality. However, based on these results, it is unknown whether this association is an epiphenomenon (since more severely ill patients have more dysglycemia) or represents a true harm from insulin and glucose infusion. Furthermore, age, severity of disease (expressed as maximum SOFA score), hypernatremia and percentage low BG measurements were all associated with mortality, as can be expected. Additionally, we analyzed patients admitted > 72 h, to ensure a cohort of the most seriously ill patients. Results were roughly comparable with the complete cohort of patients admitted > 24 h.

### Insulin

Intravenous insulin was associated with (ICU) mortality with an adjusted OR of 1.06 per 0.1 IU/kg added (95% CI 1.02–1.09). The average insulin use was 0.4 IU/kg/day (IQR 0.2–0.7). The relationship between insulin and mortality was barely affected by adjustment for history of insulin use, severity of disease, low BG measurements and other confounding factors.

Our results are in line with a post hoc analysis of the first Leuven study that showed that the daily insulin dose was a risk factor for ICU mortality (OR 1.060 per 10 IU/day added, 95% CI 1.02–1.09, *p* = 0.005). Since the ORs for mean BG exceeded those for the insulin dose, the authors concluded that glycemic control was more important than insulin dose in predicting mortality [34]. Only in patients admitted > 24 h, mean BG concentration seems to have a protective effect on mortality risk. Contrary to the limited amount of evidence in critically ill patients, in type 2 diabetes numerous epidemiological studies have showed a (dose-dependent) relation between insulin and adverse events (e.g., cardiovascular events, malignancies and mortality) [32, 42–46]. However, all these types of studies are confounded by reverse causality and confounding, especially by the exposure to other (complex) BG lowering regimens [33]. Mechanistic studies suggested that the insulin resistance of type 2 diabetes leads to a chronic state of compensatory hyperinsulinemia, which induces renal fluid retention, increased sympathetic activity and cell proliferation and differentiation [47, 48]. However, the effects of chronic hyperinsulinemia are unlikely to evoke in critical illness and data on the pathophysiological mechanism in ICU patients are scarce, although it is known that the regular insulin pathways react differently on exogenous insulin [31, 49].

Our results are in contrast to several studies in ICU patients that suggested that insulin infusion has protective effects on apoptosis after myocardial infarction [50] and induced lowering of inflammation markers [30], but those studies were not designed to assess clinical outcomes. However, the association between insulin use and mortality will be strongly influenced by the severity of disease, which will result in higher insulin resistance. We tried to correct for this by using both the APACHE IV-predicted mortality score and the maximum SOFA score, which might reflect the occurrence of organ failure during admission. In this study, we found no independent association between glucose variability and mortality, in contrast to previous studies in this and other ICUs. However, theoretically it could be argued that insulin use is one of the patient-related factors that could link glycemic variability to mortality, although there are to our knowledge no studies investigating this pathophysiological link.

### Glucose

The amount of glucose infused was low in this population with a median daily i.v. load of 0 in all patients and 1.3 g per day in patients with a length of stay > 72 h. It is therefore remarkable that after correction for confounders, intravenous glucose infusion was associated with ICU mortality with an OR of 1.01 per g/day increase (95% CI 1.00–1.01). We corrected for hypernatremia, percentage low BG and total parenteral feeding since these are the main indication for intravenous glucose and carry a worse prognosis by themselves. This result is consistent with a retrospective study in 273 long-stay (> 7 days) patients in a Dutch ICU, showing that the amount of infused glucose was independently related to ICU and hospital mortality [51]. In that study, 66 g glucose/day was infused. Moreover, the amount of infused insulin was not different between ICU survivors and non-survivors, but unlike our ICU, insulin therapy was only started above a glucose level of 12 mmol/L at discretion of the attending physician. In the Leuven study, glucose infusion was 200–260 g/day (from admission day 2 onward). Nevertheless, from these studies it remained uncertain whether infusion of exogenous glucose is also harmful when blood glucose is kept in range with the use of insulin. In contrast, several post hoc analyses of recent trials that compared early versus late parenteral nutrition found no association between glucose dose and worse outcome [52, 53].

### Limitations

In this study, we included a mixed cohort of critically ill patients, while it is plausible that there are important differences depending on the underlying illness. By using data from patients who were admitted > 72 h, we excluded the main part of the less severely ill, elective surgery, patients. However, included patients still differ in their nature and severity of disease. In general, more severely ill patients might have higher insulin needs (due to more insulin resistance) and higher glucose needs (due to conditions like liver failure, ileus, etc.). We tried to correct for this, but since this study was retrospective by design, we cannot draw conclusions on causality and the associations that were found can still be an epiphenomenon.

The standard of care in our ICU differs from the local practice in other ICUs. Here, patients do not receive parenteral feeding routinely. Almost all patients received a single dose of dexamethasone (1 mg/kg with a maximum of 100 mg) on admission to the ICU to reduce the systemic inflammatory reaction and to achieve shock reversal as part of the local practice. Glucocorticoids are known to impair insulin-mediated glucose uptake in skeletal muscles [54]. As almost all patients received steroids, we cannot assess their effect on the outcome measures. Continuous renal replacement therapy is carried out with the use of a commercially prepared bicarbonate-buffered hemofiltration replacement solution (HF32bic, Dirinco BV, Oss, the Netherlands). This replacement fluid contains one mmol/l glucose-anhydrate. Glucose can easily cross the membrane and contribute to a positive or negative glucose balance depending on patients’ characteristics [55, 56]. Since targeted BG levels in ICU patients are between 6 and 9 mmol/L, theoretically there will on average be a gradient toward glucose removal. All this together limits generalizability, and results should be interpreted with caution.

## Conclusions

In conclusion, we found arguments for an association between intravenous insulin and glucose with ICU and hospital mortality in this retrospective cohort study of critically ill patients. However, due to the single-center design with local practice, results should be interpreted with caution. Future research is needed to explore the relationship between intravenous insulin with negative ICU outcome.

## Additional file

**Additional file 1.** Characteristics of patients receiving glucose 5% (*n* = 1818).

## Abbreviations

APACHE: Acute Physiology and Chronic Health Evaluation
BG: blood glucose
ICU: intensive care unit
IQR: interquartile range
IV: intravenous
MCMC: Markov chain Monte Carlo
OR: odds ratio
PDMS: patient data management system
SD: standard deviation
STROBE: Strengthening the Reporting of Observational Studies in Epidemiology
TPV: total parenteral feeding
