# Supplementary material for: The association of intravenous insulin and glucose infusion with intensive care unit and hospital mortality: a retrospective study
Source: Ann Intensive Care. 2019 Feb 11;9:29. doi: 10.1186/s13613-019-0507-x (PMC6370891; doi:10.1186/s13613-019-0507-x)
Supplement: Supplementary file 1 — Additional file 1. Characteristics of patients receiving glucose 5% (n = 1818). [file 13613_2019_507_MOESM1_ESM.docx]

Table S1. Characteristics of patients receiving glucose 5% (n=1818).

| Variable | Glucose 5%  (n=1126) | No glucose 5%  (n=424) | P-value^a^ | Upper quintile^b^ (n=298) |
| --- | --- | --- | --- | --- |
| Sodium (mmol/L) |  |  |  |  |
| Highest during admission | 147.0 ± 5.5 | 144.0 ± 4.0 | <.001 | 148.7 ± 6.6 |
| Average during admission | 140.3 ± 3.9 | 139.0 ± 3.0 | <.001 | 140.9 ± 5.1 |
| Highest >145 | 696 (61.8) | 160 (37.7) | <.001 | 211 (70.8) |
| Average >145 | 132 (11.7) | 11 (2.6) | <.001 | 68 (22.8) |
| Data are presented as mean ± standard deviation or number (%).^a^ P-values are based on the Student’s *t* test for continuous data, and the Chi-square test (with continuity correction) or ANOVA for categorical data. APACHE, acute physiology and chronic health evaluation. | | | | |

Table S2. Characteristics of patients receiving glucose 20% (n=286).

| Variable | Glucose 20%  (n = 286) | No glucose 20%  (n = 1264) | P-value^a^ | Upper quintile^b^ (n = 71) |
| --- | --- | --- | --- | --- |
| Total parenteral feeding | 91 (31.8) | 0 | <.001 | 70 (98.6) |
| History of liver cirrhosis | 4 (1.4) | 20 (1.6) | 1.000 | 0 |
| Incidence glucose <2.2 mmol/L | 10 (3.5) | 5 (0.4) | <.0001 | 1 (1.4) |
| Incidence glucose <4.4 mmol/L | 200 (69.9) | 259 (20.5) | <.001 | 32 (45.1) |
| APACHE diagnosis |  |  |  |  |
| Sepsis | 58 (20.3) | 207 (16.4) | <.0001 | 15 (21.1) |
| Heart valve surgery | 31 (10.8) | 200 (15.8) |  | 3 (4.2) |
| After cardiac arrest | 27 (9.4) | 133 (10.5) |  | 3 (4.2) |
| Gastrointestinal | 30 (10.5) | 35 (2.8) |  | 22 (31.0) |
| Infection | 22 (7.7) | 116 (9.2) |  | 1 (1.4) |
| Perforation or obstruction | 12 (4.2) | 12 (0.9) |  | 9 (12.7) |
| Chronic cardiovascular disease | 12 (4.2) | 104 (8.2) |  | 1 (1.4) |
| Cardiovascular | 11 (3.8) | 78 (6.2) |  | 0 |
| Other | 83 (29.0) | 379 (30.0) |  | 17 (23.9) |
| Data are presented as mean ± standard deviation or number (%).^a^P-values are based on the Student’s *t* test for continuous data, and the Chi-square test (with continuity correction) or ANOVA for categorical data. APACHE, acute physiology and chronic health evaluation. | | | | |

Table S3. ICU and hospital mortality per stratum of intravenous insulin administration.

| Insulin per kg/day (IU) | ICU mortality | Adjusted OR  (95% CI)^b^ | P-  value^a^ | Hospital mortality | Adjusted OR  (95% CI)^b^ | P-  value^a^ |
| --- | --- | --- | --- | --- | --- | --- |
| No insulin | 12/162 (7.4%) | 1 |  | 21/162 (13.0%) | 1 |  |
| 0 – 0.22 | 27/347 (7.8%) | 0.72 (0.31, 1.66) | 0.441 | 42/347 (12.1%) | 0.60 (0.31, 1.15) | 0.125 |
| 0.23 – 0.42 | 35/347 (10.1%) | 0.82 (0.36, 1.91) | 0.652 | 52/347 (15.0%) | 0.69 (0.36, 1.35) | 0.283 |
| 0.43 – 0.71 | 57/347 (16.4%) | 1.56 (0.66, 3.67) | 0.310 | 77/347 (22.2%) | 1.15 (0.58, 2.29) | 0.689 |
| >0.72 | 65/347 (18.7%) | 1.88 (0.74, 4.75) | 0.184 | 90/347 (25.9%) | 1.58 (0.74, 3.38) | 0.234 |
| ICU and hospital mortality per stratum of intravenous insulin administration in IU per kg per day (n=1550). ^a^P-values as compared to the reference group (who received no intravenous insulin). ^b^Variables are chosen based on results of univariable analysis and a combination of forward and backward selection (see Table 4). CI, confidence interval; IU, OR, odds ratio. | | | | | | |

Table S4. ICU and hospital mortality per stratum of intravenous glucose administration.

| Glucose per day (g) | ICU mortality | Adjusted OR  (95% CI)^b^ | P-  value^a^ | Hospital mortality | Adjusted OR  (95% CI)^b^ | P-  value^a^ |
| --- | --- | --- | --- | --- | --- | --- |
| No glucose | 19/382 (5.0%) | 1 |  | 34/382 (8.9%) | 1 |  |
| 0 – 0.98 | 18/292 (6.2%) | 0.75 (0.37, 1.52) | 0.418 | 32/292 (11.0%) | 0.71 (0.41, 1.24) | 0.227 |
| 0.99 – 2.16 | 29/292 (9.9%) | 1.15 (0.60, 2.21) | 0.676 | 53/292 (18.2%) | 1.25 (0.75, 2.09) | 0.386 |
| 2.17 – 5.56 | 55/292 (18.8%) | 1.78 (0.96, 3.30) | 0.068 | 75/292 (25.7%) | 1.41 (0.85, 2.34) | 0.180 |
| >5.56 | 75/292 (25.7%) | 1.85 (0.94, 3.64) | 0.076 | 87/292 (29.8%) | 1.18 (0.66, 2.10) | 0.573 |
| ICU and hospital mortality per stratum of intravenous glucose administration per g per day (n=1550). ^a^P-values as compared to the reference group (who received no intravenous glucose). ^b^Variables are chosen based on results of univariable analysis and a combination of forward and backward selection (see Table 4). CI, confidence interval; IU, OR, odds ratio. | | | | | | |
